# Influence of Demographic and Socioeconomic Factors on Mental Exhaustion and Social Exclusion in the Workplace

**DOI:** 10.1192/j.eurpsy.2025.642

**Published:** 2025-08-26

**Authors:** E. D. Cindik-Herbrueggen

**Affiliations:** Psychiatry, Neuro-Psychiatric Center, München, Germany

## Abstract

**Introduction:**

Mental exhaustion and social exclusion are significant challenges in the workplace that can negatively affect well-being and job performance. Various demographic and socioeconomic factors may differently impact these phenomena. This study examines how age, gender, marital status, educational level, number of children, employment status, origin, and generation influence mental exhaustion and social exclusion in the workplace.

**Objectives:**

The primary objective of this investigation is to explore and analyze the relationships between demographic and socioeconomic factors and the dimensions of mental exhaustion and social exclusion. The study aims to identify differences and interactions among various factors, as well as to gain a deeper understanding of their impact on workplace well-being.

**Methods:**

The research was conducted by Dr. Elif Cindik, psychologist Merve Gediz, and psychology student Dicle Mutlu. A total of 73 participants were surveyed. Standardized questionnaires were used to measure mental exhaustion and social exclusion, and demographic as well as socioeconomic data were also collected. The analysis employed statistical methods such as Analysis of Variance (ANOVA), Post-Hoc Tests, Regression, and Pearson Correlation Tests to examine the relationships between the variables.

**Results:**

The tests revealed significant results in the various research areas mentioned above.

**Conclusions:**

The study highlights the complex relationships between the examined demographic and socioeconomic factors and the dimensions of mental exhaustion and social exclusion. Significantly important differences were found among older age groups, single participants, individuals with lower educational levels, and various income levels. Further research is needed to explore the causes of these differences in more detail and to develop potential intervention strategies. The findings provide valuable insights for measures aimed at improving workplace well-being.

**Disclosure of Interest:**

None Declared

